# Cytokinins – regulators of *de novo* shoot organogenesis

**DOI:** 10.3389/fpls.2023.1239133

**Published:** 2023-08-18

**Authors:** Ján Šmeringai, Petra Procházková Schrumpfová, Markéta Pernisová

**Affiliations:** ^1^ Laboratory of Functional Genomics and Proteomics, National Centre for Biomolecular Research, Faculty of Science, Masaryk University, Brno, Czechia; ^2^ Mendel Centre for Plant Genomics and Proteomics, Central European Institute of Technology, Masaryk University, Brno, Czechia

**Keywords:** cytokinin, *de novo* organogenesis, plant stem cells, shoot apical meristem, shoot regeneration

## Abstract

Plants, unlike animals, possess a unique developmental plasticity, that allows them to adapt to changing environmental conditions. A fundamental aspect of this plasticity is their ability to undergo postembryonic *de novo* organogenesis. This requires the presence of regulators that trigger and mediate specific spatiotemporal changes in developmental programs. The phytohormone cytokinin has been known as a principal regulator of plant development for more than six decades. In *de novo* shoot organogenesis and *in vitro* shoot regeneration, cytokinins are the prime candidates for the signal that determines shoot identity. Both processes of *de novo* shoot apical meristem development are accompanied by changes in gene expression, cell fate reprogramming, and the switching-on of the shoot-specific homeodomain regulator, WUSCHEL. Current understanding about the role of cytokinins in the shoot regeneration will be discussed.

## 1 Introduction

In contrast to animals, plants possess a unique developmental plasticity that enables them to adapt to changing environmental conditions. This plasticity is possible because they can undergo postembryonic *de novo* organogenesis. This strategy requires that specific spatiotemporal changes to developmental programs be made, and that is accomplished by the presence of regulators – like the phytohormones cytokinins and auxin that have been known as principal regulators of plant development for a long time (Skoog and Miller, 1957). Mutual interactions between auxin and cytokinin were shown to be involved in many developmental processes in plants (reviewed in Pernisová et al., 2011; Chandler and Werr, 2015; Schaller et al., 2015). In *de novo* organogenesis, auxin is the principal morphogen required to induce new organ onset, while cytokinins modulate the type of organogenic response. The presence of auxin alone or high auxin-to-cytokinin concentration ratio in media induces root regeneration from various plant tissues *in vitro*. On the other hand, if the auxin-to-cytokinin ratio is low, shoots are formed (Skoog and Miller, 1957; Atta et al., 2009; Pernisová et al., 2009; Sugimoto et al., 2010). Over the past few years, substantial progress has been made in understanding the molecular mechanisms underlying this ability of plants to regenerate shoot meristems. In this review, we will give a brief overview of the structure and regulations of the shoot apical meristem, before shifting our focus onto shoot regeneration and the phytohormonal regulations of the process, mainly by cytokinins.

### 1.1 Cytokinin metabolism

The source and accessibility of cytokinins to regulate plant development is largely determined by their metabolic machinery. The initial step of cytokinin biosynthesis is catalyzed by the enzyme isopentenyltransferase (IPT) and involves the transfer of a prenyl moiety from dimethylallyl diphosphate to ATP or ADP to form N^6^-isopentenyladenine (iP) ribotides (Kakimoto, 2001; Takei et al., 2001). The major initial products can be hydroxylated to *trans*-zeatin (*t*Z)-type cytokinins by the action of cytochrome P450 mono-oxygenases CYP735A1 and CYP735A2 (Takei et al., 2004). The *t*Z-types are most probably converted by zeatin reductase to DHZ-type (Martin et al., 1989; Gaudinova et al., 2005) but the corresponding gene has not yet been identified. Cytokinins of the *cis*-zeatin (*c*Z)-type arise from tRNA degradation (Kakimoto, 2001; Miyawaki et al., 2006). Direct conversion of cytokinin ribotides to active free bases is catalysed by a nucleoside 5’-monophosphate phosphoribohydrolase named LONELY GUY (LOG) which plays a pivotal role in regulating cytokinin activity during plant growth (Kurakawa et al., 2007; Kuroha et al., 2009). Free cytokinin bases and cytokinin ribosides can be converted back to ribotides by the action of adenine phosphoribosyl transferases (APT) (Lee and Moffatt, 1993; Allen et al., 2002) and adenosine kinases (ADK) (Moffatt et al., 2000), respectively. Cytokinin deactivation readily occurs via O- and N-glycosylation catalysed by uridine diphosphate glycosyltransferases (UGT) (Hou et al., 2004). Cytokinin O-glucosides can be converted to the active free bases by β-glucosidase (Brzobohaty et al., 1993). Irreversible cytokinin degradation is catalysed by the cytokinin oxidase (CKX) (Bilyeu et al., 2001; Galuszka et al., 2001), which cleaves unsaturated N^6^-side chains from *t*Z and iP-type cytokinins (Jones and Schreiber, 1997). The combined regulation of biosynthesis, modifications and degradation maintains cytokinin homeostasis, keeping phytohormone levels optimal at each stage as well as in each tissue throughout plant growth and development.

### 1.2 Cytokinin signaling

Cytokinins trigger multistep phosphorelay signaling by binding to their cognate receptors. In Arabidopsis, three receptors ARABIDOPSIS HISTIDINE KINASE AHK2, AHK3 or AHK4/WOL/CRE1 (Inoue et al., 2001; Higuchi et al., 2004; Nishimura et al., 2004) sense cytokinins via their CHASE domains (Anantharaman and Aravind, 2001). This signal is sequentially transferred to ARABIDOPSIS HISTIDINE PHOSPHOTRANSFER PROTEINS (AHPs; AHP1-5) (Hutchison et al., 2006) and then to ARABIDOPSIS RESPONSE REGULATORS (ARRs) (Suzuki et al., 1998; Tanaka et al., 2004). The one exception is AHP6, which lacks a histidine and attenuates cytokinin signaling (Mahonen et al., 2006). Type-A ARRs (ARR3-9, 15-17) represent cytokinin primary response genes and are promptly upregulated by cytokinins, while simultaneously inhibiting the cytokinin signaling pathway, thus creating a negative feedback loop (Hwang and Sheen, 2001; To et al., 2004). Type-B ARRs (ARR1, 2, 10-14, 18-21) contain a DNA binding domain and control the expression of cytokinin-regulated genes, including type-A ARRs (Sakai et al., 2000; Mason et al., 2005). A third class, type-C ARRs, consists of only two members (ARR22, 24) which are structurally related to type-A ARRs, but their transcription is not induced by cytokinins (Kiba et al., 2004). Moreover, AHPs also interact with a set of cytokinin-regulated transcription factors, the CYTOKININ RESPONSE FACTORS (CRFs) (Rashotte et al., 2006; Cutcliffe et al., 2011), thus providing additional fine-tuning of cytokinin signaling output.

## 2 Shoot apical meristem in *Arabidopsis thaliana*

### 2.1 Definition of meristems

Plants show a remarkable plasticity in their growth over their entire lifetime, which sets them apart from mammals. In plants, two apical meristematic systems localized at opposite ends of the plant body axis – the shoot apical meristem (SAM) and the root apical meristem (RAM) – are established during embryogenesis. These meristems are responsible for giving rise to the entire plant body – both the root and the areal parts. The proper functioning of meristems is dependent on the presence of a small number of undifferentiated pluripotent stem cells, which are located in specific environments called stem cell niches (reviewed in Greb and Lohmann, 2016).

### 2.2 Composition of shoot apical meristem

Stem cells of SAM are located in the central zone (CZ) at the shoot apex (Fletcher et al., 1999). Stem cells undergo very slow cell division keeping the stem cell pool constant (Laux, 2003). Daughter cells on the periphery are pushed out of the CZ to neighboring zones, where they undergo faster cell divisions and subsequently differentiate giving rise to specific plant tissues and organs (Schoof et al., 2000; Reddy et al., 2004). In Arabidopsis, CZ comprises three layers called L1, L2, and L3, where cells from each layer have a different identity. Cells in the L1 and L2 layers divide anticlinally giving rise to leaf and flower primordia. L3 cells divide anticlinally and periclinally, leading to internal tissue production. A distinct group of cells that controls CZ activity, called the organizing center (OC), is located below the CZ, and furnishes signals to block differentiation, thereby keeping stem cells undifferentiated (reviewed in Greb and Lohmann, 2016).

### 2.3 Maintenance of shoot apical meristem

**Figure 1:**
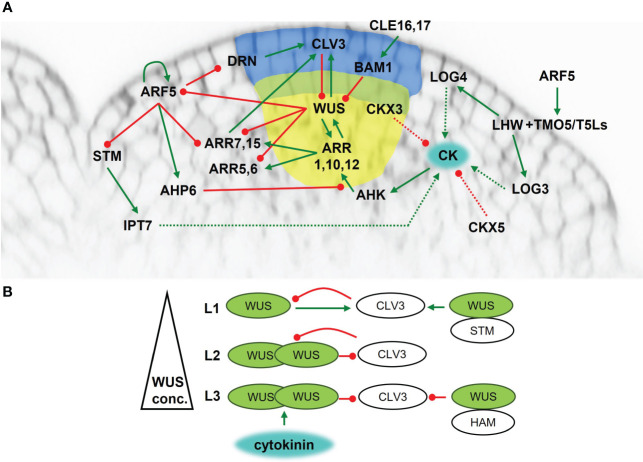
Maintenance of the shoot apical meristem of *Arabidopsis thaliana*. **(A)** Cytokinin signaling and metabolism network overview. Blue area – central zone; yellow area – organizing center; green line - positive regulation; red line – negative regulation; dashed line - metabolic pathway. **(B)** The presence of cytokinins activates WUS in the L3 layer. WUS is transported into L2 and L1, thus creating a concentration gradient. At high concentrations it forms homodimers which inhibit CLV3 activity. In the L3, WUS also forms heterodimers with HAM which strengthen the inhibitory effect on CLV3 activity. At low concentrations WUS activates CLV3 transcription. In the L1, WUS forms a heterodimer with STM, strengthening the positive regulation of CLV3.

For proper plant body development, SAM must perpetuate itself. This demands general regulation factors. Homeodomain transcription factor WUSCHEL (WUS) plays a central role in SAM maintenance as a necessary and sufficient regulator of stem cells (reviewed in Lopes et al., 2021). The *wus* mutation causes defects in the shoot meristem at all developmental stages in Arabidopsis (Laux et al., 1996; Mayer et al., 1998). In cooperation with the CLAVATA3 (CLV3) peptide, WUS preserves the stem cell niche (**Figure 1A**). Originating in the OC, WUS migrates through plasmodesmata to the CZ, where it directly activates *CLV3* expression (Yadav et al., 2011; Daum et al., 2014). The CLV3 peptide is perceived by leucine rich repeat (LRR)-based receptors, either by homomers of LRR-receptor-like kinases, such as CLAVATA1 (CLV1) (Ogawa et al., 2008), or by complexes of LRRs with membrane-bound kinases or pseudokinases, such as CLAVATA2 (CLV2) and CORYNE (CRN) (Bleckmann et al., 2009; Guo et al., 2010). CLV3 binding to dedicated receptors results in the activation of a signaling cascade that regulates WUS activity. Thus, the WUS-CLV interaction establishes a self-organizing feedback loop maintaining equilibrium between cell division and cell differentiation in the SAM (Fletcher et al., 1999; Schoof et al., 2000). Additionally, two CLV3-related peptides CLE16 and CLE17 contribute to SAM stem cell maintenance and organ production independently from CLV3. Their signal is precepted by BARELY ANY MERISTEM (BAM) and act upstream of WUS (Dao et al., 2022).

Recently, the subcellular – particularly the nucleolar versus cytoplasmic – distribution of WUS was revealed to be an important factor in stem cell niche maintenance. In L1 and L2 layers, CLV3 inhibits WUS export from the nucleus thus blocking WUS diffusion into surrounding cells. Simultaneously, CLV3 represses *WUS* expression resulting in lower WUS concentrations in the nucleus than is needed for the activation of CLV3 expression. Transport of WUS from the nucleus to the cytoplasm can be enabled by direct interaction of WUS with EXPORTIN proteins via the EAR-like domain (Plong et al., 2021). The EAR-like domain could serve as a nuclear export signal and may also be required for destabilizing WUS in the cytoplasm. Mutations in the WUS EAR-like domain leads to an enlarged SAM, which was attributed to the higher pool of cytoplasmically-stable WUS available for diffusion into adjacent cells (Rodriguez et al., 2016).

Another level of WUS action control arises from the ability of WUS to form heterodimers with other proteins (**Figure 1B**). In the L1 and L2 layers, WUS interacts with SHOOTMERISTEMLESS (STM), a transcription factor (Long et al., 1996) required for maintaining the proliferative state of the meristematic cells (Vollbrecht et al., 2000). The WUS-STM interaction strengthens its binding to the CLV3 promoter and enhances *CLV3* expression (Su et al., 2020). On the other hand, WUS can create heterodimers with proteins from HAIRY MERISTEM (HAM) family leading to the repression of *CLV3* expression in the L3 layer (Zhou et al., 2015; Zhou et al., 2018b).

### 2.4 Role of cytokinins in the regulation of shoot apical meristem

The activity of meristematic tissues is regulated by many factors, with a crucial role played by phytohormones. Cytokinins are important regulators of SAM function and maintenance, and their abundance depends on the biosynthetic machinery. One of the rate-limiting enzymes of cytokinin biosynthesis IPT7 is triggered by STM localized in SAM (Yanai et al., 2005) (**Figure 1A**). The L1 layer is thought to be the source of active cytokinins as the expression of another cytokinin biosynthetic gene LOG4 was detected there (Chickarmane et al., 2012). It is hypothesized that active cytokinins move from the L1 layer into inner cell layers, where they affect SAM function. Moreover, *LOG3* was showed to localize to developing primordia. Further, basic helix-loop-helix (bHLH) transcription factor heterodimers of TARGET OF MONOPTEROS5 (TMO5) and LONESOME HIGHWAY (LHW) subclades were suggested to act as a general regulator of cell proliferation in all meristems of Arabidopsis. Heterodimer complexes could regulate meristems due to heterodimer variations between subclade members, leading to diversification of target gene expression (Mor et al., 2022). The TMO5-LHW heterodimer directly regulates several enzymes of cytokinin metabolism (e.g., LOG3, LOG4, CKX3) (De Rybel et al., 2014; Yang et al., 2021) thus impacting endogenous cytokinin levels. In SAM, the phenotype and gene expression pattern of TMO5 and LHW subclade members suggest additional heterodimer combinations can affect LOG expression. *LOG3* and *LOG4* reporter signals are completely missing in the *lhw* single mutant suggesting that the LHW transcription factor is essential for *LOG3* and *LOG4* expression (Mor et al., 2022). Additionally, the cytokinin degrading CKX3 is expressed in a similar pattern as WUS, while CKX5 is also present in several other areas. The *ckx3 ckx5* double mutant forms larger meristems with an expanded WUS domain (Bartrina et al., 2011), probably because of higher endogenous cytokinin levels.

To be functional, cytokinins need to be “visible”. The cytokinin receptors AHK2, AHK3 and AHK4 are expressed in the inner tissues of SAM including OC, but not in the L1 and L2 layers. Therefore, it seems that it is mainly the L3 layer that is the active site for cytokinin perception (Gordon et al., 2009; Chickarmane et al., 2012). The presence of cytokinins triggers cytokinin signaling leading to the activation of type-B ARRs (**Figure 1A**). The transcription factors ARR1, ARR10 and ARR12 bind directly to the *WUS* promoter and induce *WUS* expression (Meng et al., 2017; Zubo et al., 2017). The *arr1 arr10 arr12* triple mutant forms a much smaller SAM compared to WT recalling that of the *ahk2 ahk3 ahk4* triple mutant (Ishida et al., 2008), which points to a fundamental role for ARR1, ARR10 and ARR12 in SAM maintenance. Moreover, ARR1 stabilizes WUS (Snipes et al., 2018), thus setting up positive feedback between cytokinins and WUS.

Apart from activating WUS, type-B ARRs also activate type-A ARRs (Sakai et al., 2000), thus providing negative feedback regulation of cytokinin signaling (Hwang and Sheen, 2001; To et al., 2004). ARR1, ARR2 and ARR10 are responsible for activating ARR6, with ARR2 being the most effective (Hwang and Sheen, 2001). On the other hand, several type-A ARRs, particularly ARR5, ARR6, ARR7 and ARR15, are repressed by WUS, thereby potentiating cytokinin signaling. A constitutively active form of ARR7 causes significant defects in SAM function with arrested meristem in severe cases (Leibfried et al., 2005). Consistent with this, miRNA mediated attenuation of *ARR7* and *ARR15* expression results in SAM expansion and a severe reduction of CLV3 mRNA, pointing to ARR7 and ARR15 control of SAM maintenance by regulating *CLV3* expression (Zhao et al., 2010).

It seems that local auxin accumulation is another important factor for proper functioning of ARR7 and ARR15, as revealed by their elevated transcripts in the auxin biosynthesis *yucca* (*yuc*) mutants, the auxin transport *pinformed 1* (*pin1*) mutant, or following treatment by the auxin transport inhibitor NPA. The negative regulation of ARR7 and ARR15 by auxin is partially mediated by signaling through AUXIN RESPONSE FACTOR5/MONOPTEROS (ARF5/MP) (Zhao et al., 2010). Other targets of ARF5 involved in SAM activity and maintenance have been identified in Arabidopsis, e.g., AHP6 (Besnard et al., 2014), TMOs (Schlereth et al., 2010), DORNRÖSCHEN/ENHANCER OF SHOOT REGENERATION1 (DRN/ESR1) (Cole et al., 2009), and interestingly ARF5 itself (Lau et al., 2011). The role of auxin signaling in SAM regulation and maintenance has been reviewed recently (Pernisová and Vernoux, 2021) and we will not cover this topic in more detail here.

Taken together, the overlap between cells with threshold cytokinin levels and cells competent for cytokinin perception presumably occurs at a fixed distance from the cytokinin source (L1 layer), leading to precise WUS activation, and defining the OC domain.

## 3 Two-step *in vitro* shoot regeneration via callus formation in *Arabidopsis thaliana*

### 3.1 Acquisition of meristematic identity

Shoots can be regenerated from explants in *in vitro* conditions using two approaches. During two-step regeneration, explants are first placed on a callus-inducing medium (CIM), which contains a high concentration of auxin. In the second step, the callus formed on the CIM is transferred to a shoot-inducing medium (SIM), with a high concentration of cytokinins, where shoots develop (Valvekens et al., 1988). The callus maintains some level of cell organization resembling lateral root primordium pointing to the fact that a callus is not a population of undifferentiated cells, but rather a partially differentiated tissue with features resembling the lateral root developmental program (Atta et al., 2009; Sugimoto et al., 2010). Originally, it was thought that during *de novo* organogenesis, any somatic cell can dedifferentiate and reenter the cell cycle. However, further research uncovered that organogenesis originates from populations of partially differentiated stem cells. One such population is formed of pericycle cells located adjacent to the xylem poles in roots (Dubrovsky et al., 2000; Beeckman et al., 2001). In aerial parts of the plant body, *de novo* organogenesis originates from pericycle-like cells, which are present around the vasculature of multiple organs throughout the plant body (Sugimoto et al., 2010). Initiation of founder cells is the first step in lateral root development. Acquisition of founder cell status depends on auxin accumulation in pericycle cells. The specification of founder cells is followed by nuclear polarization of two adjacent pericycle cells that divide anticlinally and asymmetrically, forming two large cells and two smaller daughter cells representing stage I of the lateral root primordium (Malamy and Benfey, 1997; Casimiro et al., 2001; Dubrovsky et al., 2001). Recent observations during the early stages of regeneration have shown that the whole process likely does not originate from a single cell, but rather from a group of cells (Subban et al., 2020).

**Figure 2:**
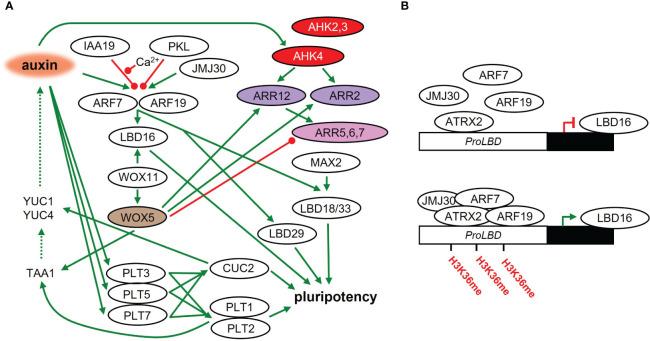
The acquisition of pluripotency in plant tissues on CIM. **(A)** Molecular regulatory network during callus formation and acquisition of pluripotency leading to shoot regeneration later on. Green line – positive regulation; red line – negative regulation; dashed line – metabolic pathway. **(B)** LBD16 activity leads to the acquisition of pluripotency on CIM. LBD16 is epigenetically activated (methylation of its promoter) by a complex composed of JMJ30, ARF7, ARF19 and ATRX2.

A local auxin maximum is established and auxin signaling is activated in the early stages of founder cell specification (**Figure 2A**). Auxin accumulation in founder cells leads to the degradation of the AUXIN/INDOLE-3-ACETIC ACID INDUCIBLE28 (AUX/IAA28) repressor, which controls the founder cell-specifying GATA23 transcription factor (De Rybel et al., 2010). Auxin also controls the division of founder cells by degrading SOLITARY ROOT/INDOLE 3 ACETIC ACID INDUCIBLE14 (SLR/IAA14), which acts as a repressor of ARF7 and ARF19. The repression of ARF7 and ARF19 is also dependent on PICKLE/SUPRESSOR OF SLR2 (PKL/SSL2). Following SLR/IAA14 degradation, ARF7 and ARF19 positively regulate the expression of LATERAL ORGAN BOUNDARIES-DOMAIN29/ASYMMETRIC LEAVES2-LIKE16 (LBD29/ASL16), LBD16/ASL18 and LBD18/ASL20 (Fukaki et al., 2002; Okushima et al., 2005; Fukaki et al., 2006), that are necessary for the initiation of lateral roots (Okushima et al., 2007; Lee et al., 2009). LBD induction promotes cell cycle progression through the G1 – S checkpoint (Feng et al., 2012). The process of callus formation seems also to be positively regulated by MORE AUXILARY GROWTH2 (MAX2) (Li et al., 2019). In the *max2* mutant, LBD33 is less upregulated on CIM medium (Temmerman et al., 2022). LBD33 forms a heterodimer with a LBD18, and this is responsible for the induction of pericycle cell cycle activation via E2Fa transcription factor (Okushima et al., 2007; Berckmans et al., 2011).

Ca^2+^-mediated signaling plays an important role in auxin-controlled initiation of callus formation. CALMODULIN IQ-MOTIF CONTAINING PROTEIN (CaM-IQM) physically interacts with IAA19 in a Ca^2+^-dependent manner, which then destabilizes the repressive interaction of IAA19 with ARF7, promoting callus formation via LBD activity (Zhang et al., 2022). The impact of Cam-IQM on other IAAs or ARFs is unclear so far.

LBD16 is controlled by WUCHEL-RELATED HOMEOBOX 11 (WOX11), which is activated shortly after the acquisition of root primordium identity by founder cells (Liu et al., 2014). WOX11 also activates another member of the WOX family, WOX5 (Hu and Xu, 2016), broadly expressed in the subepidermal layer of each callus type (Sugimoto et al., 2010). WOX5 was originally identified as a WUS homolog in SAM and is analogically responsible for the maintenance of the root quiescent center in the stem cell niche (Sarkar et al., 2007; Pi et al., 2015). Moreover, WOX5 directly affects auxin levels in cooperation with the AP2-family transcription factors PLETHORA1 (PLT1) and PLT2 by activating the auxin biosynthetic gene TRYPTOPHAN AMINOTRANSFERASE OF ARABIDOPSIS 1 (TAA1) (Zhai and Xu, 2021).

During CIM cultivation, the expression of PLT3, PLT5, and PLT7 is rapidly induced. They control PLT1 and PLT2, leading to the establishment of a pluripotent callus (Kareem et al., 2015). Analysis of the gene regulatory network uncovered PLT3 as a critical node in shoot regeneration (Ikeuchi et al., 2018). Meristem development is also dependent on the PLT1 gradient (Galinha et al., 2007), which indicates the existence of a fine-tuned transcriptional regulatory mechanism. In addition, the activity of PLT3, PLT5, and PLT7 regulates the shoot-promoting factor CUPSHAPED COTYLEDON2 (CUC2) (Kareem et al., 2015), which was shown to promote adventitious shoot formation (Daimon et al., 2003). *CUC* genes play an important role in the regulation of auxin abundance by controlling the auxin biosynthetic genes *YUC1* and *YUC4* (Yamada et al., 2022). CUC2 promotes meristem formation by activating the cell wall loosening enzyme (XTH9), whose activity is triggered in cells surrounding the meristem progenitor. This results in a mechanical conflict, triggering cell polarity in the progenitors and the subsequent promotion of meristem formation (Varapparambath et al., 2022).

Callus formation is dependent on cytokinin perception by the receptors AHK2, AHK3 and AHK4. The corresponding mutants exhibit reduced callus size and greening (Li et al., 2019). Moreover, the presence of auxin in CIM leads to strong local up-regulation of AHK4, which predestines future cytokinin-induced WUS expression (Gordon et al., 2009). Auxin signaling is therefore partially responsible for the sensitivity of the whole system to cytokinins. Further cytokinin signal transduction also plays an important role in the acquisition of pluripotency in the callus. The type-B ARR2 and ARR12 interact with the root-specific WOX5 that promotes cytokinin sensitivity of the system (Zhai and Xu, 2021). It seems that type-B ARRs also affect the recalcitrancy of plant tissues. *ARR18* expression was much lower in recalcitrant Arabidopsis lines than in highly regenerative ones (Lall et al., 2004). Furthermore, WOX5 also represses type-A ARRs, negative regulators of cytokinin signaling. Overexpression of WOX5 inhibits the expression of *ARR5*, *ARR6* and *ARR7*. Complementarily, the transcript level of *ARR5* was higher in the *wox5* mutant on both CIM and SIM (Zhai and Xu, 2021; Lee et al., 2022). The loss of ARR7 function resulted in strong induction of callus formation, which points to an inhibitory role for ARR7 in the acquisition of pluripotency (Buechel et al., 2010). Preincubation on CIM is necessary for the expression and proper function of ARR15, a direct target of ARR2 (Che et al., 2008). Thus, a proper balance between various up- and down-regulators of cytokinin signaling is crucial for the acquisition of pluripotency.

Information about changes in endogenous cytokinin levels in Arabidopsis during processes leading to *de novo* shoot regeneration *in vitro* is rather scarce. Arabidopsis hypocotyls cultivated on medium supplemented with kinetin at concentrations of 0, 300 and 1000 ng/ml had comparable dynamics of endogenous *t*Z-type cytokinins levels, whereas the levels of iP-type cytokinins strongly increased on media containing a high kinetin concentration (1000 ng/ml) (Pernisova et al., 2018). This seems to suggest a possible role for iP-type cytokinins in the development of *de novo* shoot apical meristems *in vitro*.

#### 3.1.1 Epigenetic regulation of pluripotency acquisition

The importance of epigenetic regulation of *de novo* organogenesis has become increasingly clear over the past few years. Research has shown that histone acetyl-transferase/GENERAL CONTROL NONDEREPRESSIBLE (HAG1/CGN5) is essential for *de novo* shoot regeneration already during callus development. It catalyzes histone acetylation at meristem gene loci of many *de novo* organogenesis regulators such as WOX5, WOX14, PLT1 or PLT2, thus forming an epigenetic platform for their transcriptional activation (Kim et al., 2018).

Methylation also works as a frequent mechanism for epigenetic regulation of regeneration. One of the pathways is mediated by the JUMONJI C DOMAIN-CONTAINING PROTEIN 30 (JMJ 30), which stimulates the callus formation by binding to ARF7 and ARF15 activating the LBD genes, by removing methyl groups from H3K9me3, especially at the LBD16 and LBD29 promoters (**Figure 2B**). JMJ30-ARF complex further recruits ARABIDOPSIS TRITHORAX-RELATED 2 (ATXR2), a histone lysine methyltransferase promoting the accumulation of H3 histones trimethylated on lysine 36 (H3K36me3), which interacts with ARF7 and ARF19 and increases H3K36me3 accumulation on LBD promoters (Lee et al., 2017; Lee et al., 2018), and is thus responsible for stimulation of callus formation.

HISTONE THREE RELATED (H3.15) is active in processes connected to plant regeneration. It is distinguished from other Arabidopsis histones by the absence of lysine residue 27 that is trimethylated by POLYCOMB REPRESSIVE COMPLEX 2 (PRC2). Its presence results in transcriptional derepression of downstream genes such as WOX11. H3.15 expression increases after wounding of plant tissue (Yan et al., 2020), but any possible role in *de novo* organogenesis remains unclear.

#### 3.1.2 Control of pluripotency acquisition regulated by miRNA

The effect of miRNA molecules on the acquisition of pluripotency and callus formation from explants is also significant. The Arabidopsis miR160 inhibits callus formation via the cleavage of ARF10 and ARF16 mRNAs, which defines the spatial expression patterns of regulators such as WOX5 and PLTs (Wang et al., 2005; Ding and Friml, 2010). Cytokinin signaling is also affected by miR160 via repression of ARF10, a negative regulator of ARR15. Activity of miR160 results in the inhibition of callus initiation by enhancing ARR15 expression (Liu et al., 2016). Thus, miR160 may play an important role in the regulation of both auxin as well as cytokinin signaling. Cytokinin responses are modulated also by the activity of miR156, which represses SQUAMOSA PROMOTER BINDING PROTEIN-LIKE (SPL) genes, defining an age-dependent pathway controlling *de novo* shoot organogenesis. The repression of SPL leads to the abolition of *de novo* shoot regenerative capacity by attenuating cytokinin responses via the modulation of ARR1 (Barrera-Rojas et al., 2020).

### 3.2 Meristem identity respecification

To regenerate a shoot, cells first acquire competence for organogenesis on CIM with a high concentration of auxin. The calli are then exposed to a high concentration of cytokinins on SIM, which promotes the acquisition of shoot meristem properties and subsequent shoot organogenesis (Christianson and Warnick, 1983). A rapid downregulation of root-specific markers occurs shortly after transferring the callus to SIM. Root specific *WOX5* expression in the middle cell layer of callus is gradually reduced within 48 hours after transfer to SIM (Zhai and Xu, 2021). Accordingly, the *ProWOX5:GFP* signal is downregulated in a cytokinin concentration-dependent manner (Pernisova et al., 2018). Expression of *LBD16*, activated by WOX11, is also reduced after transfer to SIM (Liu et al., 2018b). Shoot-specific genes are activated at the same time as root-specific genes become attenuated. Of the transcription factors involved in shoot regeneration, WUS and STM play a key role in the initiation of shoot organogenesis (Hibara et al., 2003; Gordon et al., 2009; Chatfield et al., 2013). The shoot-specific *ProWUS:tdTomato* signal was detectable as soon as three days on cytokinin-rich media (Pernisova et al., 2018). Accordingly, *wus* mutants fail to regenerate shoots *in vitro* (Zhang et al., 2017b). Another member of the WOX family, WOX14 can be an important factor in shoot regeneration. A WOX14 overexpressor was able to regenerate shoots on cytokinin free medium. The *ProWOX14:GUS* reporter line exhibited *WOX14* activity on SIM, but not on CIM (Wang et al., 2023a). However, the molecular mechanisms behind these results have yet to be uncovered.

**Figure 3:**
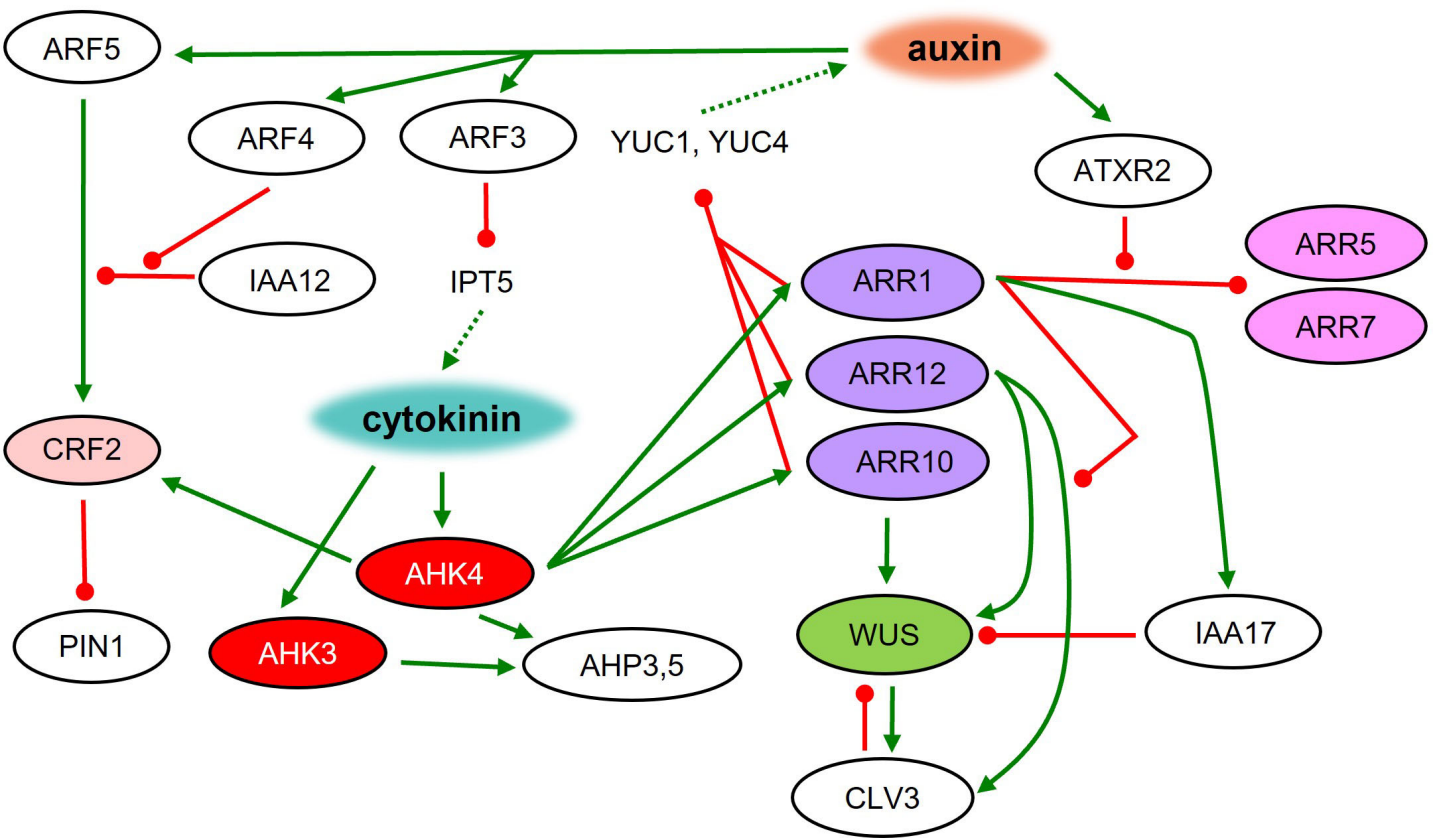
Root to shoot meristem identity respecification on SIM. Molecular regulatory network during shoot regeneration on cytokinin-rich medium. Green line – positive regulation; red line – negative regulation; dashed line – metabolic pathway.

The activity of general regulators requires the interplay and achievement of a fine balance of auxin and cytokinin function at several levels. For successful shoot regeneration, auxin must first trigger a program leading to the establishment of pluripotent status, while the presence of cytokinins modulates the organogenic response leading to shoot formation. Cytokinins quickly downregulate auxin signaling in a concentration-dependent manner, leading to a loss of root identity (Pernisova et al., 2018). The perturbation of auxin signaling was revealed to be crucial for the future induction of shoot regeneration on SIM (Ohbayashi et al., 2022). Auxin signaling can be affected by reducing the source, e.g., by decreased biosynthesis or disrupted transport. Cytokinin signaling suppresses the expression of auxin biosynthetic genes in the central region of the forming meristem, which can lead to decreased auxin levels (**Figure 3**). The transcription factors ARR1, ARR10, and ARR12 interact with YUC promoters leading to the inhibition of *YUC1* and *YUC4* expression (Meng et al., 2017). Application of auxin biosynthesis inhibitors or polar auxin transport inhibitors on CIM enhanced subsequent shoot formation on SIM (Ohbayashi et al., 2022). Also, cytokinins affect auxin distribution by regulating the expression of auxin transporters from the PIN FORMED (PIN) family (Pernisová et al., 2009). CRFs acting downstream of cytokinin perception, participate in shoot regeneration as well. Shoot formation is induced in *crf2* mutants and diminished in *crf5* mutants during *in vitro* regeneration (Rashotte et al., 2006). The expression of *CRF2* is dependent on a crucial auxin signaling component ARF5 (Ckurshumova et al., 2014), thus connecting cytokinin and auxin signaling pathways. The activity of ARF5 is negatively regulated by IAA12, and in the presence of auxin, ARF5 repression is compromised by the presence of ARF4, which competes with ARF5 in binding to IAA12. ARF4 compromises the level of free IAA12, thus maintaining the activity of ARF5 (Zhang et al., 2021). Another member of auxin signaling, ARF3 acts as a negative regulator of *de novo* organ regeneration. It binds directly to the promoter of *IPT5*, which disrupts the cytokinin biosynthesis pathway (Cheng et al., 2012). ARF3 binding to the *IPT5* promoter requires high auxin concentration and is thus attenuated on SIM, leading to the activation of cytokinin biosynthesis. Taken together, fine-tuning between the effects of auxins and cytokinins is fundamental for proper plant organ regeneration.

The regulation of SAM formation during shoot regeneration requires WUS as a central player. WUS represents one of the first shoot-specific markers active in SAM-forming cells (Pernisova et al., 2018). Loss of WUS function leads to a severe disruption of shoot regeneration (Gordon et al., 2007; Chatfield et al., 2013). In the later stages of shoot regeneration, WUS activity can be observed only in the regions where the AHK4 cytokinin receptor was previously expressed during incubation on CIM (Gordon et al., 2009). Areas with an active AHK4 during CIM incubation also define the future positions of auxin transport protein PIN1 upregulated during SIM incubation (Gordon et al., 2007; Atta et al., 2009; Gordon et al., 2009). Application of the histidine kinase inhibitor TSCA during the first four days of SIM incubation leads to the inhibition of shoot regeneration in Arabidopsis. This inhibition seems to be at least partially connected to the disruption of cytokinin signaling via AHK4, AHK3, AHP3 and AHP5 (Lardon et al., 2022). Overall, AHK4 seems to be pivotal in the cytokinin-mediated regulation of organ identity affecting shoot-specific WUS as well as root-specific WOX5 activity (Pernisova et al., 2018).

Other members of the cytokinin signaling pathway – response regulators – are also involved in shoot regeneration. *arr1* mutants generate more shoots than the wild type. It was shown that ARR1 binds to the *IAA17* promoter, inducing its expression during SIM cultivation, leading to the indirect WUS inhibition and disruption of shoot regeneration (Liu et al., 2020). Another cytokinin-related transcription factor ARR12 promotes the *CLV3* expression. Additionally, ARR1 causes repression of *CLV3* in an ARR12 dependent manner. It was revealed that ARR1 and ARR12 compete in binding to the *CLV3* promoter, which regulates its activity (Liu et al., 2020). Moreover, ARR12 positively regulates *WUS* by binding to its promoter and activating expression (Dai et al., 2017). Thus, ARR12 seems to be a critical player in shoot stem cell niche maintenance and shoot regeneration. Additionally, the ectopic expression of *ARR2* and *ARR11* gives rise to *in vitro* shoot regeneration in the absence of exogenous cytokinins (Hwang and Sheen, 2001; Imamura et al., 2003) as seen also with cytokinin-independent1 (CKI1) (Kakimoto, 1996).

On the other hand, the activity of type-A ARRs (negative regulators of cytokinin signaling) usually results in a reduction of shoot regeneration (Buechel et al., 2010). ARR1 binds directly to the promoters of *type-A ARR5* and *ARR7*, which causes their repression. On SIM, ATXR2 is responsible for H3K336me3 deposition at *ARR5* and *ARR7* promoters thus disrupting ARR1 binding and activating *type-A ARRs* (Lee et al., 2021). ARR8 seems to also have an impact on shoot regeneration capacity. ARR8 overexpression abolishes SAM formation (Osakabe et al., 2002).

WUS and CLV3 activity and subsequent shoot regeneration are dependent also on abiotic factors, such as light or temperature. It was shown that ELONGATED HYPOCOTYL (HY5) a transcription factor of light signaling is responsible for inhibition of *CLV3* and *WUS* by direct interaction with their promoter. Indirect inhibition of WUS and CLV3 is achieved through the interaction between HY5 and ARR12 (Dai et al., 2022). Temperature affects shoot regeneration competency by depleting the histone variant H2A.Z which acts as a repressor of *de novo* shoot organogenesis and is present at 17°C but removed at 27°C (Lambolez et al., 2022).

ALTERED MERISTEM PROGRAM 1 (AMP1) is a putative Glu carboxypeptidase present in Arabidopsis. Defective AMP1 results in the formation of enlarged shoot meristems, a larger stem cell pool and a higher rate of leaf formation (Huang et al., 2015). AMP1 affects stem cell niche patterning by controlling the downstream HD-ZIPIII/RAP2.6 transcription factor module. RAP2.6L is under direct transcriptional control of HD-ZIP III transcription factors via their binding to the *RAP2.6L* promoter. The presence of HD-ZIP III is elevated in the *amp1* mutant. AMP1, therefore, seems to limit SAM activity via miRNA-dependent control of HD-ZIP III transcription factors and subsequent RAP2.6L activity (Yang et al., 2018). The regulatory pathways of HD-ZIP III activity also comprise auxin activity. A novel chemical inhibitor of polar auxin transport ZIC2 (LITTLE ZIPPER3 inducing compound) promotes shoot regeneration and subsequent RAP2.6L elevation in an HD-ZIP III-dependent manner (Yang et al., 2022). Moreover, previous research suggests that oxygen also works as a signal, as its spatial distribution affects the proteolysis of LITTLE ZIPPER 2 (ZPR2), controlling the activity of HD-ZIP III transcription factors (Weits et al., 2019). These findings together lift the lid on the exquisite complexity of the HD-ZIP III transcription factor regulatory network in the process of shoot regeneration.

#### 3.2.1 Epigenetic regulation of shoot regeneration

Acquisition of shoot identity in the callus is an important step during *de novo* shoot organogenesis. Recent research indicates that epigenetic regulations are necessary for this process, especially in WUS activity induction. LYSINE-SPECIFIC DEMETHYLASE 1-LIKE 3 (LDL3) demethylates lysine 4 of histone H3 (H3K4me2), which leads to the reduction of the H3K4me2 tag and subsequent acquisition of shoot regeneration competency. H3K4me2 may be responsible for the control of *WUS* activity together with other tags such as H3K9ac, H3K4me3, and H3K9me2 (Li et al., 2011; Ishihara et al., 2019). Moreover, cytokinins launch division-dependent removal of the repressive H3K27me3 tag from *WUS* promoter (Zhang et al., 2017b). In this inducible state, *WUS* can be activated by type-B ARRs, for instance ARR10, directly targeting *WUS* (Zubo et al., 2017). The deposition of H3K27me3 by PRC2 (Zhou et al., 2018a) may be coordinated also by Telomeric repeat binding (TRB) proteins (Schrumpfová et al., 2016; Kusová et al., 2023). TRB proteins also recruit H3K4 demethylase JMJ14 implicated in the regulation of *WUS* expression (Li et al., 2011; Wang et al., 2023b).

On the other hand, shoot regeneration can be repressed by the function of DNA METHYLTRANSFERASE (MET1) by inhibiting *WUS* expression. MET1 activity can be promoted by a cell cycle regulator – transcription factor E2FA (Liu et al., 2018a). The ability of cells to undergo cell division is a prerequisite for shoot regeneration (Tamaki et al., 2009). Whole-genome bisulfite sequencing of the *met1-3* mutant uncovered the dependence of plant regeneration on CG methylation of target genes including CRYPTOCHROME1 (CRY1) and CRY2. CRY1 is responsible for the regulation of cytokinin signaling connected to the induction of shoot regeneration mainly via type-B ARRs (Shim et al., 2021).

A recent study revealed the role of HISTONE DEACETYLASE 19 (HDA19) in *de novo* shoot regeneration. HDA19 is a member of the histone deacetylase family, which deacetylates histones at CUC2 and DRN/ESR1 loci. In the *hda19* mutant line, histones at the CUC2 and DRN/ESR1 loci are in a hyperacetylated state, leading to their excessive expression and consequent reduced shoot regeneration capacity. In WT, CUC2 and DRN/ESR1 are active only in distinct cell groups, which will gain shoot forming identity. However, in *hda19 CUC2* and *DRN/ESR1* are continually expressed throughout the explant and no localized expression patterns are produced. It seems that the expression patterns of *CUC2* and *DRN/ESR1* are crucial for future *de novo* shoot regeneration (Temman et al., 2023).

#### 3.2.2 miRNA impact on shoot regeneration

During *in vitro* shoot regeneration, miRNA molecules play an important role in various pathways regulating successful shoot formation. miRNA molecules are also responsible for age-regulated developmental timing, which affects the ability of plant tissue to regenerate shoots. A high concentration of miR156 is responsible for the prolongation of juvenile phases by targeting the SPL group of proteins (Xu et al., 2016). Plant regenerative capacity decreases with increasing age. It is a consequence of the gradual increase in miR156 levels targeting SPLs, which attenuate the cytokinin response by binding to type-B ARRs (Zhang et al., 2015). miRNA molecules also modulate the cytokinin response. The small RNA methyltransferase HUA ENHANCER1 (HEN1) is responsible for miR319 production. Mutations in HEN1 lead to a strong reduction of miR319 level and subsequent increase in its transcription factor targets – TEOSINTE BRANCHED 1 CYCLOIDEA AND PCF TRANSCRIPTION FACTOR 3 (TCP3) and TCP4. They are responsible for the activation of *ARR16* expression, resulting in the inhibition of shoot regeneration (Yang et al., 2020). Regulation via miRNA also impacts auxin signaling. On SIM, overexpression of miR393a causes the attenuation of auxin signaling through its interaction with the auxin receptor TRANSPORT INHIBITOR RESPONSE1 (TIR1) resulting in TIR1 inhibition, disruption of auxin signaling, and subsequent defects in shoot regeneration (Wang et al., 2018). Some miRNAs can intervene in both cytokinin and auxin signaling. Based on its presence in calli incapable of shoot regeneration, miR160 was discovered as a negative regulator of SAM formation. miR160 targets and inhibits ARF10, a positive factor in SAM formation (Qiao et al., 2012; Qiao and Xiang, 2013).

Stem cell niche organization, among others, is regulated by the HD-ZIP III group of transcription factors. Their activity is partially regulated by cytokinin signaling. Type-B ARRs (ARR1, ARR2, ARR10, and ARR12) physically interact with HD ZIP III and these complexes activate WUS expression, which leads to shoot regeneration (Zhang et al., 2017a). Other than WUS-mediated stem cell niche maintenance, the activity of HD-ZIP III seems to also control a WUS-independent pathway. HD-ZIP III TFs act as repressors of this pathway, and their inactivity in the *wus-1* mutant background, leads to a normal functional SAM with normal cell layering and meristem morphology (Lee and Clark, 2015). The activity of HD-ZIP III TFs is regulated at the posttranscriptional level through miR165/166 because HD-ZIP III TFs have nearly perfect complementarity with the sequence located near the 3’end of the fourth exon and the 5’end of the fifth exon with miR165/166 (Mallory et al., 2004; Zhang and Zhang, 2012). The accumulation of miR165/166 is negatively affected by the activity of an EIF2C protein ARGONAUT 10 (AGO10). *ago10* mutants produce higher number of SAMs, and the stem-cell markers WUS, CLV3, and STM are strongly expressed (Xue et al., 2017).

## 4 Direct conversion of root primordia to shoot meristems in *Arabidopsis thaliana*

**Figure 4:**
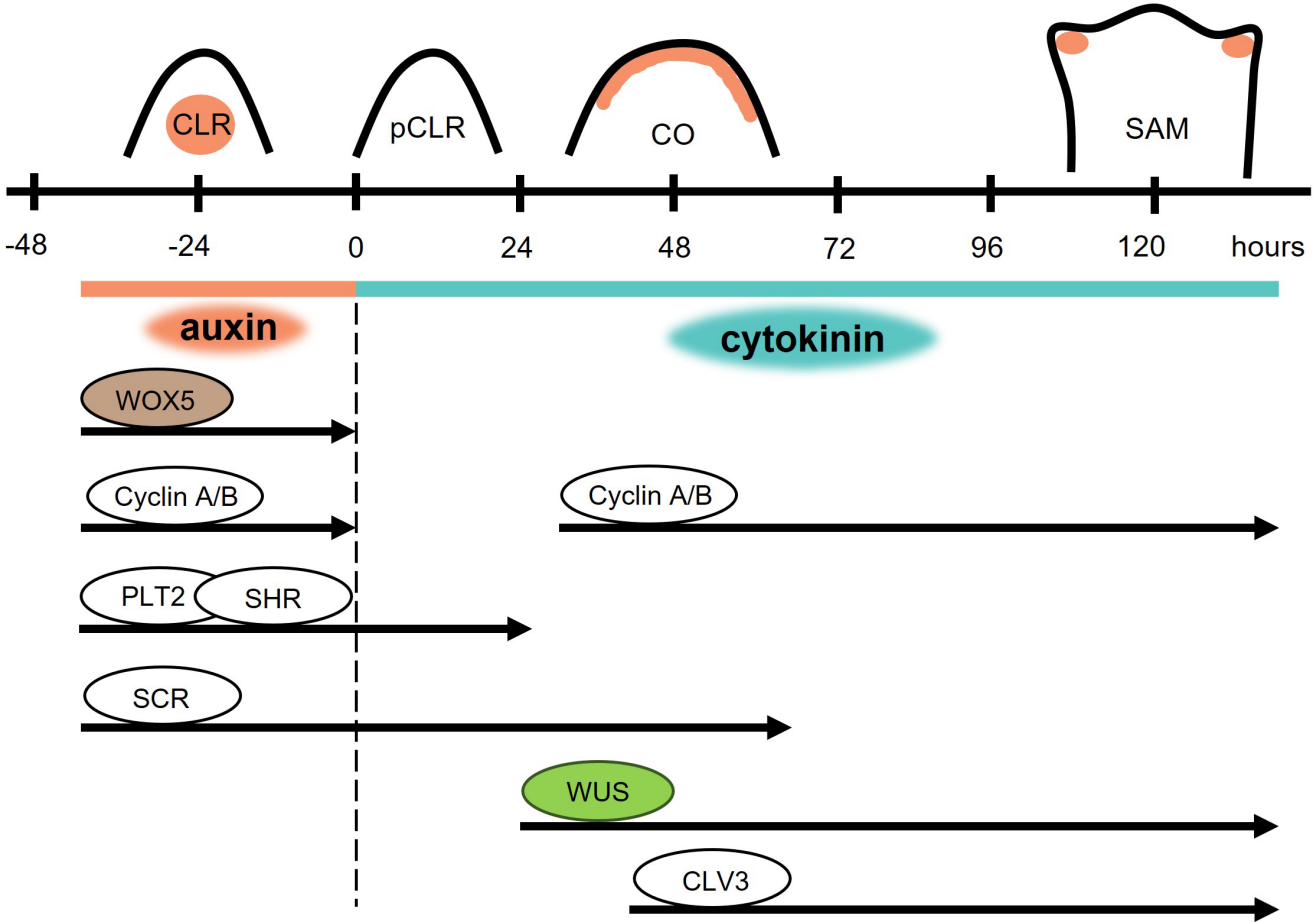
Direct conversion of root primordia to shoot meristems. High auxin concentration triggers the formation of a competent lateral root (CLR). After the transfer to cytokinin rich medium (dashed black line), cell division is attenuated in the paused competent lateral root (pCLR). The cell cycle is paused for 24 hours and expression of root-specific *WOX5* is terminated. The activity of other root identity markers lasts for 24 hours after transfer for PLT2 and SHR, and 63 hours for SCR. The shoot identity markers WUS and CLV3 are detected after 19 and 32 hours, respectively. 48 hours after transfer to cytokinin rich medium, conversion of meristematic identity takes place in the converting organ (CO). 120 hours after transfer, a shoot apical meristem (SAM) is formed.

Apart from indirect shoot regeneration via cultivation on CIM and SIM, through the intermediate step of callus formation, direct conversion of lateral root primordia (LRP) to shoot apical meristems is also possible (Chatfield et al., 2013; Kareem et al., 2016; Rosspopoff et al., 2017). During this process, roots are cultivated on a medium with a high concentration of auxin to activate LRP development (Chatfield et al., 2013) (**Figure 4**) that comprises eight developmental stages (Malamy and Benfey, 1997). Subsequently, roots with LRPs are transferred to a medium containing a high concentration of cytokinins, usually of the iP-type. The whole process occurs without any signs of dedifferentiation or callus formation and is called transdifferentiation. The transition from LRP to SAM is possible only during a narrow developmental window, in which the LRPs are in developmental stages VI – VII. The organogenic programs in these tissues are remarkably plastic and their identity can be redirected multiple times by the addition of cytokinins and auxins (Rosspopoff et al., 2017). Moreover, the highest responsiveness of LRPs to conversion into shoots were seen in stages II and III (Kareem et al., 2016), whereas stages V and VI were considered the least sensitive to cytokinins (Bielach et al., 2012). Despite these discrepancies, the developmental stage of the LRP is a very important factor, influencing the success of the transition.

Few morphological changes can be observed during the first 24 hours following the transfer of explants to the cytokinin-rich medium. This is a sign of rapidly decreased mitotic activity (Rosspopoff et al., 2017); transcriptome analysis revealed a reduced expression of A- and B-type cyclins and cyclin-dependent kinases (**Figure 4**) - pointing to a reduction in cell division (Chatfield et al., 2013). During the second and third day, mitotic activity is restored in the provasculature and in the upper half of the primordium, creating a transitioning organ. After three days, an early shoot promeristem is formed on top of the transitioning organ and leaf primordia appear after four to five days (Rosspopoff et al., 2017).

The early changes in this direct switch of primordium identity are connected to changes in auxin signaling caused by the presence of cytokinins. Shortly after the exposure to cytokinins, auxin levels are rapidly reduced and the auxin transporter PIN1 is cleared out of the plasma membrane. Auxin levels are restored on the top of the transitioning organ with PIN1 active at the plasma membranes of L2 and L3 layers. In the fully structured shoot meristem, auxin is concentrated in the leaf primordia (Rosspopoff et al., 2017).

24 hours after cytokinin exposure, root specific PLT2 and SHR expression is undetectable (Rosspopoff et al., 2017) (**Figure 4**). On the other hand, SCR was detectable even after 63 hours of cytokinin treatment, but its activity was attenuated afterwards (Kareem et al., 2016). Shortly after cytokinin exposure, activity of the root-specific WOX5 rapidly decreases, and after a brief mitotic inactivity the same cells possessing root identity switch to the expression of shoot identity markers such as WUS, CLV3, and STM. The WUS reporter was first visible after 19 hours on cytokinin-rich medium and was followed by CLV3 after 32 – 48 hours (Chatfield et al., 2013). After the restoration of mitotic activity, WUS, CLV3, and STM activity is restricted to the apex of the transitioning organ and the domains of their expression overlap. When the promeristem is formed, CLV3 and WUS domains resemble their typical organization in SAM, in which CLV3 marks the central zone comprising stem cells and WUS the organizing centre. However, it seems that only WUS expression is not sufficient for direct *in vitro* shoot regeneration, because WUS expression was detected also in stage V of LRP development, in which the ability to form shoots was not assessed (Rosspopoff et al., 2017). Altogether, facts support the theory that neither dedifferentiation nor the formation of a new stem cell niche is necessary for *de novo* shoot organogenesis, and the same cells can be transdifferentiated.

## 5 Perspectives

*In vitro* shoot regeneration has been used as a reliable tool in plant propagation where seedling-based propagation is either not feasible or not appropriate. Although it has been known for more than six decades that cytokinins play a critical and irreplaceable role in shoot regeneration, we are only now beginning to uncover the detailed molecular and physiological mechanisms that are behind this role. For instance, although plant propagation protocols have most often relied on callus-based regeneration to successfully propagate viable plants, recent discoveries have proved that this two-step process is not actually necessary. One-step shoot regeneration can enable faster and more efficient production of plant material and has the potential to speed up the whole process. To achieve this goal, a better understanding of the underlying molecular mechanisms is necessary, especially the role of cytokinin signaling and metabolism, as also the interactions with other factors. Such interactions – especially with auxin – are exquisitely complex and many aspects of the process remain to be clarified. Possible differences in the molecular mechanisms during one-step and two-step *in vitro* shoot regeneration may also be uncovered, which would lead to greater comprehension of the whole process leading to shoot regeneration.

Cytokinins possess very complex signaling and especially metabolic pathways. Distinguishing the role of individual cytokinin metabolic enzymes taking part in shoot regeneration is complicated by redundant biosynthetic pathways leading to the production of active cytokinins. Moreover, the impact of exogenous cytokinins presented in cultivation media is not clear yet. A broad spectrum of gene editing techniques is necessary to uncover particular branches of the complex network that are behind shoot regeneration. Omics, single cell and subcellular approaches can help elucidate the fine balance present in such a complex network in even more detail.

Comprehending the central role of cytokinins in shoot apical meristem maintenance and *in vitro* shoot regeneration can provide a highly valuable tool enabling better control over the process. This better control can then be applied to come up with more efficient protocols for plant production. Moreover, such knowledge could be used for fine-tuning plant micropropagation and regeneration protocols. This could then mean higher efficiency *in vitro* regeneration of recalcitrant species or genetic lines with reduced *in vitro* viability. The combination of these two limitations is one of the most significant obstacles for crop improvement, because the successful transformation of crops in desirable gene targets is being hampered by the inability to establish viable tissue culture. Overcoming these obstacles may be highly beneficial for both research and commercial goals.

## Author contributions

All authors listed have made a substantial, direct, and intellectual contribution to the work and approved it for publication.
